# Supplementary material for: Hepatocyte Growth Factor Regulates the miR-206-HDAC4 Cascade to Control Neurogenic Muscle Atrophy following Surgical Denervation in Mice
Source: Mol Ther Nucleic Acids. 2018 Jul 6;12:568–77. doi: 10.1016/j.omtn.2018.06.013 (PMC6077135; doi:10.1016/j.omtn.2018.06.013)
Supplement: Document S1. Figures S1–S3 and Table S1 [file mmc1.pdf]

OMTN, Volume *12*

Supplemental Information

Hepatocyte Growth Factor Regulates the miR-206-HDAC4 Cascade to Control Neurogenic Muscle Atrophy following Surgical Denervation in Mice

Wooshik Choi, Junghun Lee, Jaeman Lee, Kyeong Ryang Ko, and Sunyoung Kim

Figure S1, related to Figure 3

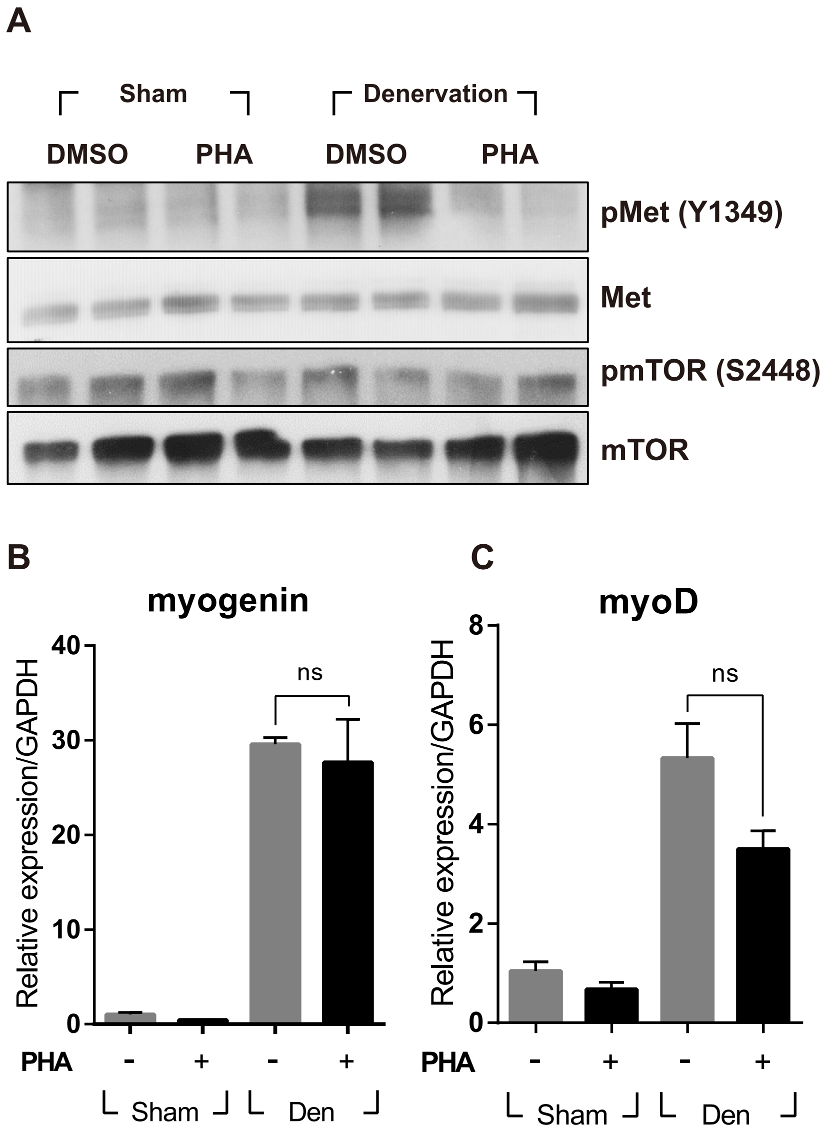

Figure S1. Effect of c-met inhibitor, PHA-665752 on c-met, mTOR phosphorylation and E-box transcription factors. After denervation by sciatic nerve transection, mice were intraperitoneally injected with 20 mg/kg of PHA-665752 on a daily basis until sacrificed. Three days later, TA muscles were prepared and total RNAs and proteins were isolated followed by RT-qPCR and Western blot respectively. (A) Effect on total and phosphorylated c-met, mTOR protein. (B) Effect on myogenin RNA. (C) Effect on myoD RNA. Values were normalized to GAPDH. ns=not significant. n=4 per group.

Figure S2, related to Figure 4

A

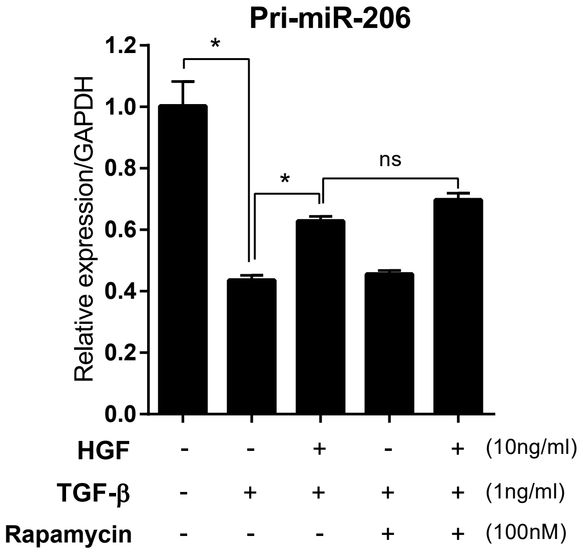

Figure S2. Effect of mTOR in HGF-mediated regulation of miR-206 expression in C2C12 cells.
C2C12 cells were plated and cultured in differentiation medium in the presence or absence of recombinant TGF-β and HGF proteins. Total RNAs were prepared and analyzed for miR-206 primary transcript. (A) Effect of mTOR inhibitor on the HGF-mediated regulation of pri-miR-206 transcript expression. Values were normalized to GAPDH. ns=not significant. *$p<0.05$, (unpaired student's t test), n=2 per group.

Figure S3, related to Figure 5

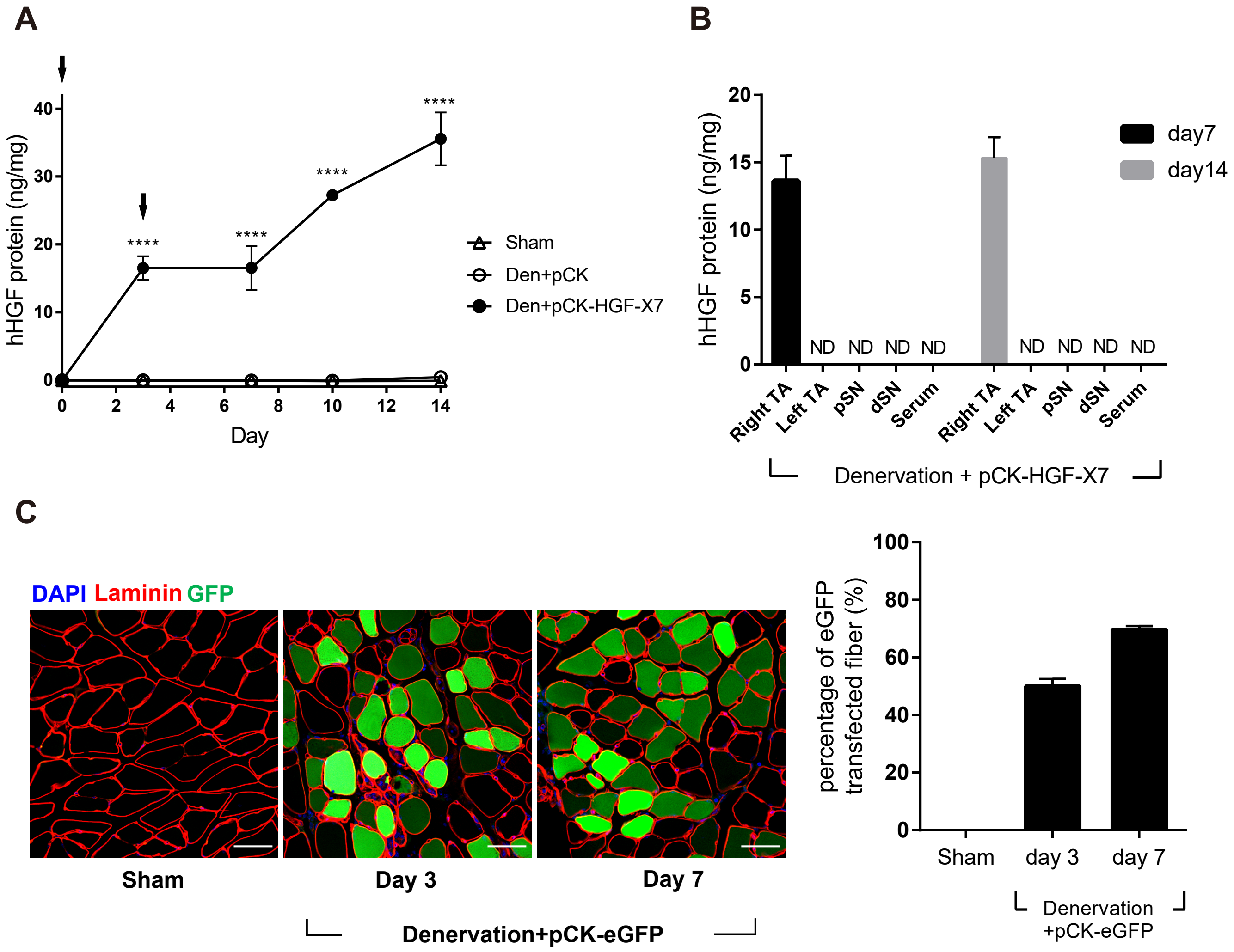

Figure S3. hHGF expression by pCK-HGF-X7 in denervated mice.
pCK-HGF-X7 was i.m. injected at the time(day 0) of sciatic nerve transection followed by one more injection on day 7. (A) Expression kinetics of the hHGF protein after denervation. Two arrows indicate i.m. injection of pCK-HGF-X7 on days 0 and 7. The muscle was isolated at 3, 7, 10, and 14 days after denervation, and total proteins were analyzed by ELISA to measure the protein level of human HGF. *$p<0.05$, **$p<0.01$, ***$p<0.001$, ****$p<0.0001$ versus pCK-treated muscle (unpaired student's t test), n=4 per group. (B) Expression kinetics of hHGF protein in TA, proximal and distal sciatic nerve, and serum. ND=not detected, n=2 per group. (C) Transfection efficiency of pCK-eGFP in denervated muscle. pCK-eGFP was i.m. injected at the time of sciatic nerve transection. TAs were costained with GFP and the membrane marker laminin (red). n=3 per group. Scale bars=50μm.

Table S1

A

Primers	sequence
mHGF_F	5'-ATCCACGATGTTCATGAGAG-3'
mHGF_R	5'-GCTGACTGCATTTCTCATTC-3'
MURF1_F	5′-TGCCTGGAGATGTTTACCAAGC-3′
MURF1_R	5′-AAACGACCTCCAGACATGGACA-3'
Atrogin-1_F	5′-AAGGCTGTTGGAGCTGATAGCA-3′
Atrogin-1_R	5′-CACCCACATGTTAATGTTGCCC-3′
Pri-miR-206_F	5'-ACCCAGTGCCCTGTGTTCCCA-3'
Pri-miR-206_R	5'-AGCGCCTCTTCTCGGTTTCCCT-3'
HDAC4_F	5'-CACACCTCTTGGAGGGTACAA-3'
HDAC4_R	5'-AGCCCATCAGCTGTTTTGTC-3'
MyoD_F	5'-CCACTCCGGGACATAGACTTG-3'
MyoD_R	5'-AAAAGCGCAGGTCTGGTGAG-3'
Myogenin_F	5'-GAGACATCCCCCTATTTCTACCA-3'
Myogenin_R	5'-GCTCAGTCCGCTCATAGCC-3'
GAPDH_F	5'-CTGGAAAGCTGTGGCGTGAT-3'
GAPDH_R	5'-CCAGGCGGCACGTCAGATCC-3'

B

Antibody	host	company	catalog	Notes
pMet (Y1349)	rabbit	Cell signaling	#3133	WB, 1:500
Met	rabbit	Sigma Aldrich	SAB4300599	WB, 1:2000
HDAC4	mouse	Cell signaling	#5392	WB, 1:1000
pSmad3 (S423/425)	rabbit	Cell signaling	#9520	WB, 1:1000
Smad3	rabbit	Cell signaling	#9523	WB, 1:1000
Laminin	rabbit	Sigma Aldrich	L9393	IHC, 1:1000
GFP	mouse	Millipore	MAB3580	IHC, 1:500

Table S1. Primers and antibodies used in this study.
(A) Primer information for RT-qPCR analysis. (B) Antibody information for Western blot hybridization and Immunohistochemistry.
