# Supplementary material for: Hepatocyte Growth Factor Regulates the miR-206-HDAC4 Cascade to Control Neurogenic Muscle Atrophy following Surgical Denervation in Mice
Source: Mol Ther Nucleic Acids. 2018 Jul 6;12:568–77. doi: 10.1016/j.omtn.2018.06.013 (PMC6077135; doi:10.1016/j.omtn.2018.06.013)
Supplement: Document S2. Article plus Supplemental Information [file mmc2.pdf]

Molecular Therapy
Nucleic Acids
Original Article

Hepatocyte Growth Factor Regulates the miR-206-HDAC4 Cascade to Control Neurogenic Muscle Atrophy following Surgical Denervation in Mice

Wooshik Choi,[1] Junghun Lee,[2] Jaeman Lee,[1] Kyeong Ryang Ko,[1,2] and Sunyoung Kim[1,2]

[1]School of Biological Sciences, Seoul National University, Seoul 08826, Korea; [2]ViroMed Co., Ltd., Bldg. 203, Seoul National University, Seoul 08826, Korea

Hepatocyte growth factor (HGF) has been well characterized for its roles in the migration of muscle progenitors during embryogenesis and the differentiation of muscle stem cells, but its function in adult neurogenic muscle atrophic conditions is poorly understood. Here we investigated whether HGF/c-met signaling has any effects on muscle-atrophic conditions. It was found that HGF expression was upregulated in skeletal muscle tissue following surgical denervation and in hSOD1-G93A transgenic mice showing severe muscle loss. Pharmacological inhibition of the c-met receptor decreased the expression level of pri-miR-206, enhanced that of HDAC4 and atrogenes, and resulted in increased muscle atrophy. In C2C12 cells, HGF inhibited phosphorylation of Smad3 and relieved TGF-β-mediated suppression of miR-206 expression via JNK. When extra HGF was exogenously provided through intramuscular injection of plasmid DNA expressing HGF, the extent of muscle atrophy was reduced, and the levels of all affected biochemical markers were changed accordingly, including those of primary and mature miR-206, HDAC4, and various atrogenes. Taken together, our finding suggested that HGF might play an important role in regard to neurogenic muscle atrophy and that HGF might be used as a platform to develop therapeutic agents for neuromuscular disorders.

INTRODUCTION

Skeletal muscle is a highly dynamic tissue that can vary in size, structure, and contractile force under different conditions. Innervation of the motor neuron provides various trophic factors to the target muscle that are essential to maintain skeletal muscle function. One of the pathological hallmarks of motor neuron diseases, such as amyotrophic lateral sclerosis (ALS) or poliomyelitis, is deterioration of muscle innervation. In these diseases, the skeletal muscle undergoes severe physiological changes, such as debilitating muscle loss because of the deficiency in neural input. Loss of nerve supply to muscle fiber could activate the muscle atrophy program, including activation of ubiquitin-dependent proteasomal or autophagosomal lysis of the muscle components.[1] The muscle-specific E3-ubiquitin ligases MuRF1 and Atrogin-1 are known to be responsible for proteasomal degradation of muscle. Histone deacetylase 4 (HDAC4) has been reported to positively regulate the expression of these E3-ubiquitin ligases via two independent mechanisms, especially in neurogenic muscle atrophy.[2,3]

MicroRNAs (miRNAs) are single-stranded 21- to 22-nt noncoding RNAs that can control gene expression via a post-transcriptional mechanism. Specific miRNAs have recently been discovered as critical regulatory factors controlling skeletal muscle metabolism, including muscle differentiation and homeostasis. For example, miR-206, a member of muscle-enriched miRNAs (myo-miR), is known to facilitate muscle differentiation by regulating the expression of myogenic regulatory factors *in vitro*[4,5] and *in vivo*.[6] It was recently shown that miR-206 could delay the progression of ALS by suppressing the expression of HDAC4 and, thereby, promoting regeneration of the neuromuscular synapse, suggesting that miR-206 might affect the course of the neurogenic muscle-atrophic condition.[7]

Hepatocyte growth factor (HGF) was first discovered as a potent mitogen for hepatocytes and later found to also contain mitogenic, morphogenic, angiogenic, anti-apoptotic, and anti-fibrotic activities.[8–12] It is well known that interaction of HGF with its cellular receptor, c-met, turns on a variety of signaling pathways, such as Stat3, Erk, and Akt, depending on the cell types. In skeletal muscle, HGF is known to be secreted by activated muscle stem cells (also known as satellite cells) *in vivo*[13,14] as well as *in vitro*.[15,16] Upon muscle injury, HGF activates muscle stem cells that reside in muscle fiber, leading to regeneration of damaged muscle.[14,17] Exogenously added recombinant HGF protein has been shown to ameliorate pathological conditions in mouse models for hypoxia-induced muscle atrophy[18], polymyositis and dermatomyositis.[19] It has been reported that HGF can promote the survival of motor neurons *in vitro*[20] and that HGF overexpression might attenuate the death of motor neurons and axon degeneration in ALS mice.[21] Despite its interesting biological characteristics, the role of HGF regarding muscles under denervation conditions remains poorly understood.

Here we report that the role of HGF is partially compensational in neurogenic muscle atrophy. HGF expression was upregulated following surgical denervation. When mice were treated with

Received 10 April 2018; accepted 25 June 2018;
https://doi.org/10.1016/j.omtn.2018.06.013.

Correspondence: Sunyoung Kim, School of Biological Sciences, Seoul National University, Building 504, Gwanak-Gu, Seoul 08826, Korea.
E-mail: sunyoung@snu.ac.kr

 © 2018 The Author(s).
This is an open access article under the CC BY license (http://creativecommons.org/licenses/by/4.0/).

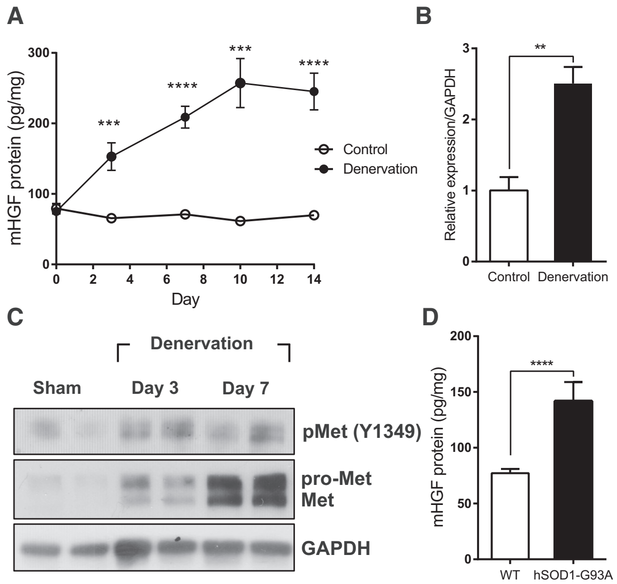

Figure 1. Expression Kinetics of HGF in Denervated Muscle

(A) Expression kinetics of HGF protein after denervation. The muscle was isolated 3, 7, 10, and 14 days after denervation, and total proteins were analyzed by ELISA to measure the protein level of HGF. ***$p < 0.001$, ****$p < 0.0001$ versus control muscle (unpaired Student's t test), n = 4 per group. (B) Change in the RNA level of HGF after denervation. RNAs were prepared from TA muscles 3 days after denervation, followed by qRT-PCR. The values were normalized to glyceraldehyde-3-phosphate dehydrogenase (GAPDH). **$p < 0.01$ (unpaired student's t test). (C) Expression kinetics of c-met and phosphorylated c-met proteins in denervated TA muscle. Muscle was isolated on days 3 and 7, and total proteins were prepared, followed by western blot using specific antibodies to total or phosphorylated c-met. Each lane represents a sample from an individual mouse. Two representative results are shown. Two independent experiments were performed (n = 4), and similar results were obtained. (D) Comparison of the HGF protein level in TA muscles between wild-type (WT) and 150-day-old hSOD1-G93A transgenic mice. The TA muscle was isolated, and total proteins were analyzed by ELISA to measure the protein level of HGF. ****$p < 0.0001$ versus WT mice (unpaired Student's t test), n = 6 per group. All data are represented as mean ± SEM.

PHA-665752, an inhibitor of the c-met receptor, muscle atrophy was exacerbated. Consistently, the expression level of HDAC4 was further increased, whereas the opposite was the case for miR-206. HGF overexpression by intramuscular (i.m.) injection of a plasmid expression vector slowed down the progression of muscle atrophy. Data from C2C12 cell culture experiments indicated that HGF regulated the expression of miR-206 by suppressing transforming growth factor β (TGF-β)-mediated phosphorylation of Smad3. Taken together, our data suggest that HGF might be used as a platform for developing therapeutic agents to treat neurogenic muscle atrophy.

RESULTS

HGF/c-met Signaling Was Upregulated in Denervated Muscle

To investigate the possible involvement of HGF in neurogenic muscle atrophy, a sciatic nerve transection model, in which irreversible damage was made to the nerve by cutting the sciatic nerve, was used. Denervation was induced by severing the sciatic nerve of a 10-week-old C57BL/6 mouse, and total proteins were prepared from the tibialis anterior (TA) muscle of the injury site at appropriate time points, followed by ELISA. The basal level of HGF protein on the control side was maintained at 50–80 pg/mg of total cellular protein in the TA muscle. After denervation, the level of HGF protein on the ipsilateral side was rapidly increased, reaching a plateau at approximately 250 pg/mg of total cellular protein on day 10 (Figure 1A). A similar magnitude of RNA induction was observed after denervation, as measured by qRT-PCR (Figure 1B). These data suggest that HGF expression was induced by 3- to 5-fold after denervation at both the RNA and protein levels compared with the normal, uninjured situation.

C-met is the only known receptor for HGF. When HGF is expressed, its receptor, c-met, becomes activated by phosphorylation. Therefore, the level and content of c-met protein were analyzed after nerve injury in the same sciatic nerve transection model. Total proteins were prepared from the TA muscle, followed by western blot using antibodies to total c-met or the phosphorylated form (Figure 1C). After denervation, the level of total c-met protein rapidly increased, and the phosphorylated form of c-met protein was also upregulated in the denervated muscle.

The effect of denervation on HGF expression was also measured in hSOD1-G93A transgenic mice, a widely used model for ALS. These mice overexpress the mutated superoxide dismutase (SOD1) protein, resulting in motor neuron death and severe muscle wasting throughout the entire body.[22] Total proteins were prepared from the TA muscle of hSOD1-G93A transgenic mice on day 150 after birth, when the muscle atrophy progressed severely, and the HGF protein level was measured using ELISA. Wild-type mice produced 70-–80 pg/mg of HGF in the TA muscle. In hSOD1-G93A transgenic mice, the amount of the HGF protein was higher by approximately 2-fold (Figure 1D).

Inhibition of c-met Signaling Aggravated Neurogenic Muscle Atrophy

It was tested whether denervation-induced expression of HGF played a pathological or compensational role using an inhibitor specific to the c-met receptor, PHA-665752. After sciatic nerve transection, mice were intraperitoneally (i.p.) injected with PHA-665752 on a daily basis. Treatment with PHA-665752 effectively suppressed c-met phosphorylation in denervated muscle (Figure S1A). Ten days later, the TA muscle mass from vehicle (DMSO)-treated animals was found to be reduced by 24% ± 2%, from 50.1 1.1 mg to 38.1 ± 1.0 mg, compared with the sham-operated group, whereas PHA-665752-treated mice showed a larger reduction, by 34% ± 3% (Figure 2A). The skeletal muscle cross-section was analyzed by H&E staining of the TA muscle. In vehicle-treated mice, muscle fiber size was decreased by 41% ± 1%, from 1,671 ± 128 μm^2 to 972 ± 14 μm^2, compared with the sham-operated animals. In PHA-665752-treated mice, it was further reduced, by 51% ± 1%, compared with the sham-operated group (Figure 2B). These data indicate that inhibition of c-met signaling could worsen muscle mass and cross-sectional area during neurogenic muscle atrophy, suggesting that HGF works as part of the compensatory system.

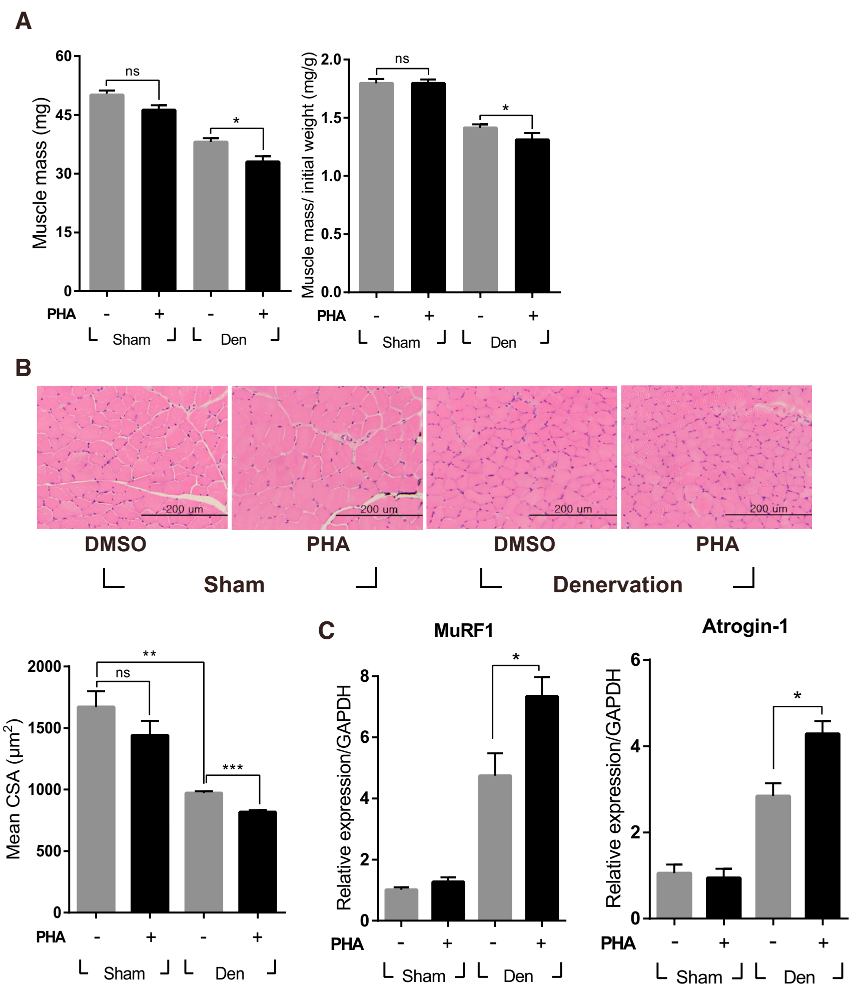

Figure 2. Effect of the c-met Inhibitor PHA-665752 on Muscle Atrophy in the Sciatic Nerve Transection Model

After sciatic nerve transection, mice were injected i.p. with 20 mg/kg of PHA-665752 on a daily basis until sacrifice. (A) Effect on muscle weight. The graph on the left shows actual weight, whereas, on the right, muscle mass was normalized with the initial weight of mice. Den, denervation; PHA, PHA-665752; ns, not significant. $^{*}p < 0.05$ (one-way ANOVA), n = 5 per group. (B) The effect on the cross-sectional area (CSA) of TA muscle was analyzed 10 days after denervation. At least 300 muscle fiber areas were counted per sample. Mean CSA is indicated in the graph. $^{**}p < 0.05$, $^{***}p < 0.01$ (one-way ANOVA). Scale bars, 200 μm. (C) Effect on the expression of MuRF1 and Atrogin-1. The RNA level of two genes was determined by real-time qRT-PCR using TA muscles isolated 3 days after denervation. $^{*}p < 0.05$ (one-way ANOVA), n = 4 per group. All data are represented as mean ± SEM.

MuRF1 and Atrogin-1 are involved in proteasomal degradation of muscle components, and their expression is highly increased at the RNA level after denervation. The sciatic nerve was severed to induce denervation of the TA muscle. Three days later, RNAs were isolated from TA muscles of mice when the RNA level of MuRF1 and Atrogin-1 was greatly induced. In animals treated with PHA-665752, the expression of MuRF1 and Atrogin-1 was even further increased (Figure 2C). In sham-operated animals, PHA-665752 did not have significant effects on either gene. These data suggest that the HGF/c-met signaling pathway might counteract the process of neurogenic muscle atrophy by controlling the expression of genes involved in muscle breakdown.

c-met Signaling Controls the miR-206-HDAC4 Cascade

Because HDAC4 is a key player in the regulation of MuRF1 and Atrogin-1 during neurogenic muscle atrophy,[2] the effect of PHA-665752 on the denervation-mediated increase of HDAC4 expression was studied by qRT-PCR and western blot. As shown in Figure 3A, the RNA level of HDAC4 was highly increased after denervation, whereas treatment with PHA-665752 did not have any effect. The protein level of HDAC4 showed a similar pattern (a sharp increase after denervation), whereas treatment with PHA-665752 always gave a small but highly reproducible increase in the level of HDAC4 compared with the untreated but denervated animals (Figure 3B; compare lanes 5 and 6 with lanes 7 and 8). These data indicate that HDAC4 expression might be controlled at the post-transcriptional level.

HDAC4 expression has previously been shown to be regulated by miR-206 under muscle-atrophic conditions.[7] To test whether miR-206 expression was affected by c-met signaling, the level of primary miR-206 transcript was analyzed in TA muscles by qRT-PCR 3 days after nerve transection in the presence or absence of PHA-665752 administration. Denervation markedly increased the level of pri-miR-206. When animals were treated with PHA-665752, however, the level of pri-miR-206 transcript was reduced in both sham and denervated mice (Figure 3C). The magnitude of reduction was approximately 2-fold in both cases. Taken together, these data suggest that c-met signaling can downregulate HDAC4 expression by upregulating miR-206, not only under denervation but also in the uninjured situation.

miR-206 expression is known to be controlled by two different pathways; one is E-box transcription factors, including myoD and myogenin,[23,24] and the other is TGF-β signaling.[25,26] We found that treatment with PHA-665752 had little or no effect on the former (Figures S1B and S1C). TGF-β is highly induced in denervated muscle and participates in developing pathological conditions.[25] The antagonistic relationship between HGF and TGF-β signaling has already been reported under fibrotic conditions.[27,28] Therefore, it was tested whether HGF regulates the expression of miRNA by interacting with TGF-β signaling. Because TGF-β signaling is already known

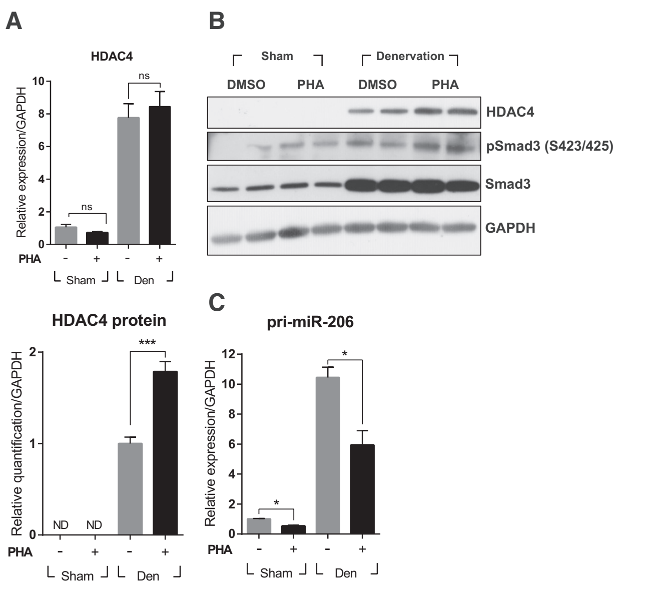

Figure 3. Effect of the c-met Inhibitor PHA-665752 on the miR-206-HDAC4 Cascade

After denervation by sciatic nerve transection, mice were injected i.p. with 20 mg/kg of PHA-665752 on a daily basis until sacrifice. Three days later, TA muscles were prepared, and total RNAs and proteins were isolated, followed by qRT-PCR or western blot. (A) Effect on HDAC4 RNA. n = 4 per group. (B) Effect on HDAC4 and total and phosphorylated Smad3 protein. This presents two representative results from two independent experiments, with the total number of mice being 4. The graph shows the result of quantification of HDAC4 protein. Values were normalized to GAPDH. ***$p < 0.001$ (unpaired student's t test). (C) Effect on the miR-206 primary transcript. *$p < 0.05$ (one-way ANOVA), n = 4 per group. ND, not detected. All data are represented as mean ± SEM. See also Figure S1.

to downregulate the expression of miR-206 through its canonical pathway, Smad2/3 signaling,[26] the effect of PHA-665752 on Smad3 phosphorylation was tested. Total proteins were prepared from TA muscles, followed by western blot using antibodies detecting Smad3 or its phosphorylated form. As expected, denervation significantly increased the level of total and phosphorylated Smad3 (Figure 3B; compare lanes 1 and 2 with lanes 5 and 6). However, when animals were treated with PHA-665752, Smad3 phosphorylation was even more increased in both sham and denervated mice (Figure 3B; compare lanes 5 and 6 with lanes 7 and 8). These data indicate that HGF/c-met signaling might regulate the expression of miR-206 through the Smad3-dependent pathway.

HGF Regulates miRNA-206 Expression by Suppressing TGF-β Signaling

To understand the mechanism(s) underlying the effect of HGF at the molecular and cellular levels *in vitro*, C2C12, a murine myoblast cell line, was used. Cells were differentiated to myotubes by changing the medium to DMEM supplemented with 2% horse serum. Four days later, cells were treated with various concentrations of recombinant human HGF (hHGF) protein in the presence of 1 ng/ml of recombinant TGF-β for 24 hr. When differentiated C2C12 myotubes were treated with TGF-β only, the expression level of pri-miR-206 was reduced to about 40% compared with the untreated control. Cotreatment with 10 ng/ml HGF inhibited a TGF-β-mediated decrease in the level of pri-miR-206 transcript, resulting in a 1.5-fold increase compared with the TGF-β-only group (Figure 4A). Similar patterns were observed when the expression level of mature miR-206 was measured (Figure 4B). The level of miR-206 was not affected by HGF in the absence of TGF-β, suggesting that HGF might upregulate the expression of miR-206 by suppressing TGF-β signaling.

Next, the effect of HGF on Smad3 phosphorylation was tested. C2C12 cells were pretreated with various concentrations of hHGF protein for 30 min, followed by incubation with 2 ng/ml TGF-β for an additional 30 min. Treatment with TGF-β increased the level of phosphorylated Smad3 up to 4-fold (Figure 4C; compare lane 1 with lane 5). The presence of HGF lowered it in a dose-dependent manner, whereas the level of total Smad3 remained unchanged (Figure 4C). These results indicate that HGF might control the expression of miR-206 by inhibiting Smad3 phosphorylation induced by TGF-β.

It is well known that HGF/c-met signaling utilizes downstream effectors such as Erk1/2, p38, JNK, Akt, and mTOR, to induce various cellular responses. It was tested which downstream effectors of HGF/c-met signaling would be involved in the suppression of Smad3 phosphorylation. C2C12 cells were pretreated with pharmacological inhibitors of Erk1/2, p38, JNK, Akt, and mTOR for 30 min, followed by treatment with 10 ng/ml of hHGF for 30 min and then by incubation with 2 ng/ml of TGF-β for an additional 30 min. Again, HGF inhibited TGF-β-induced Smad3 phosphorylation (Figure 4D; compare lane 2 with lane 3). Among different inhibitors, SP600125, an inhibitor of JNK, seems to be the only one that can rescue the HGF-mediated suppression of Smad3 phosphorylation (Figure 4D; compare lane 3 with lane 6). Consistent with these data, treatment with SP600125 significantly reduced the effect of HGF on the pri-miR-206 expression suppressed by TGF-β (Figure 4E). Taken together, these data suggest that JNK might act as a downstream signal of the HGF/c-met pathway to inhibit Smad3 phosphorylation.

Exogenous Introduction of HGF Alleviates Neurogenic Muscle Atrophy

Based on the above data indicating a positive role(s) of HGF in muscle atrophy, we tested the effects of exogenous addition of HGF in the same model. Because HGF has a very short half-life, less than 5 min in serum, the use of recombinant HGF protein for this purpose was not thought to be a viable approach.[29] In the following experiments, we delivered HGF using a plasmid DNA expression vector. pCK-HGF-X7 (or VM202) is a plasmid designed to express two isoforms of hHGF, HGF723 (or dHGF) and HGF728 (or cHGF), at high levels *in vivo*,[30–32] and it has been used in a variety of clinical studies and animal models.[30–34]

Denervation was induced by severing the sciatic nerve of a 10-week-old C57BL/6 mouse, and 100 μg of pCK-HGF-X7 or pCK control

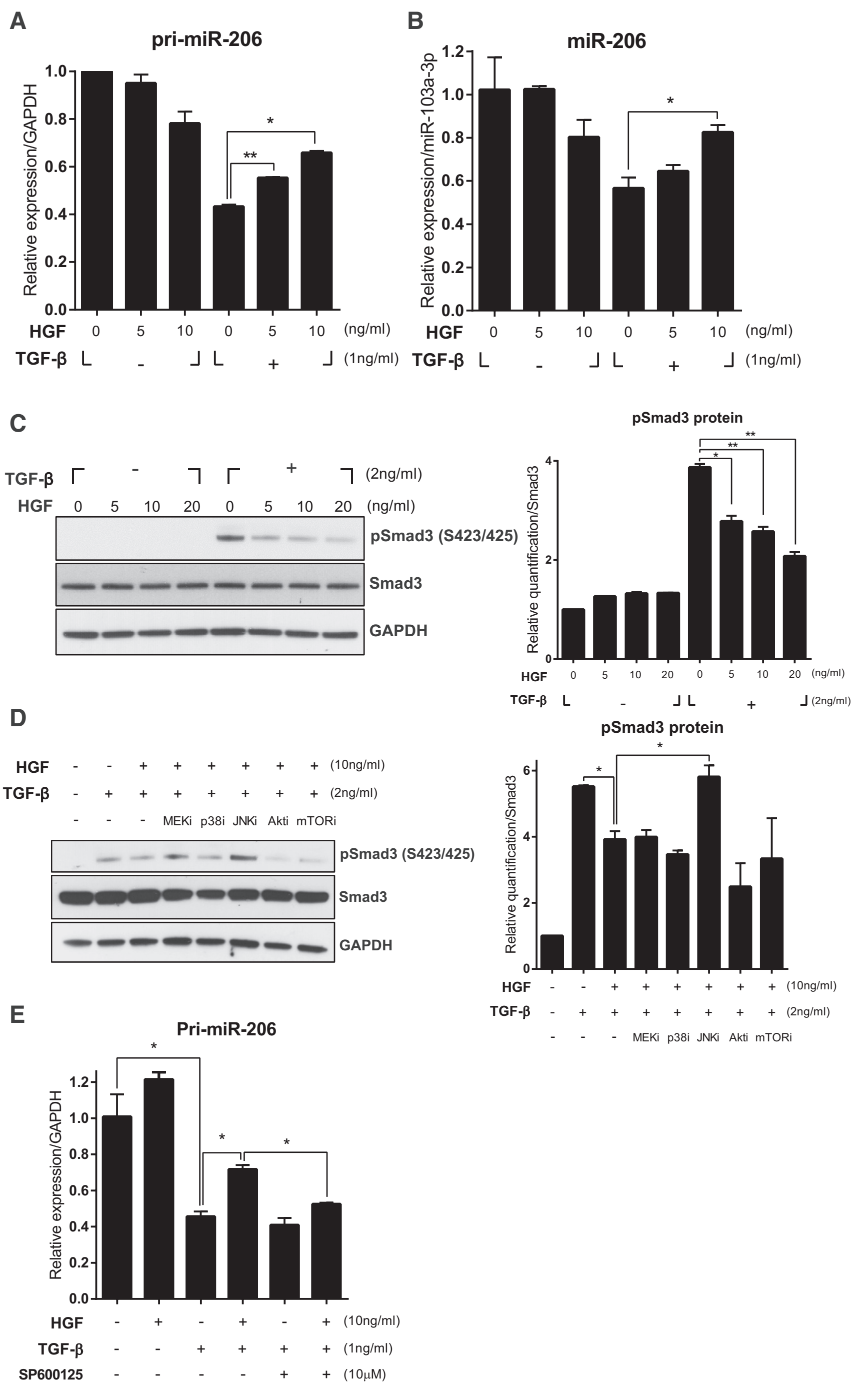

Figure 4. Effect of Recombinant HGF Protein on miR-206 and Smad3 in C2C12 Cells

C2C12 cells were plated and then cultured in differentiation medium in the presence or absence of recombinant TGF-β and HGF proteins. Total RNAs and proteins were prepared and analyzed for miR-206 and Smad3 by qRT-PCR and western blot, respectively. For the western blot, two independent experiments were performed, and one representative result is shown. The graph displays the result of the protein band quantification. (A) Effect on the pri-miR-206 transcript. Values were normalized to GAPDH. $*p < 0.05$, $**p < 0.01$ (unpaired Student's t test), n = 3 per group. (B) Effect on mature miR-206. Values were normalized to miR-103a-3p. $*p < 0.05$, (unpaired Student's t test), n = 3 per group. (C) Effect of HGF on Smad3 phosphorylation. The graph shows the result of protein band quantification. Values were normalized to total Smad3. $*p < 0.05$, $**p < 0.01$ (unpaired Student's t test). (D) Effect of various chemical inhibitors on HGF-mediated suppression of phosphorylated Smad3. Values were normalized to total Smad3. $*p < 0.05$ (unpaired Student's t test). (E) Effect of the JNK inhibitor on HGF-mediated regulation of pri-miR-206 transcript expression. Values were normalized to GAPDH. $*p < 0.05$, (unpaired Student's t test), n = 3 per group. All data are represented as mean ± SEM. See also Figure S2.

vector lacking the HGF sequence was administered i.m. into the ipsilateral TA muscle, followed by a second injection 7 days later. The *in vivo* protein expression kinetics of this plasmid have been well established previously;[30–32] the protein level of hHGF produced from pCK-HGF-X7 gradually increases upon injection, reaching a peak (about 30 ng/mg) 7 days after the first injection and then steadily decreases before returning to the control level after approximately 2 weeks.[30,31] The hHGF protein is detectable within 5–10 mm from an injection needle point (K.R.K., unpublished data).

The TA muscle was isolated and quantitated at different time points after denervation. As shown in Figure 5A, in denervated mice injected with the pCK control vector, muscle mass was decreased by 32% and 42% on days 10 and 14, respectively. When mice were injected with pCK-HGF-X7, the reduction of muscle weight was slowed down, to 21% and 34% compared with the control, on days 10 and 14, respectively.

The muscle cross-section was analyzed by H&E staining to measure muscle fiber size 10 days after denervation. In pCK-treated animals, muscle fiber size was decreased by 61% ± 1% compared with the sham-operated group, from 1,750 ± 173 μm^2 to 688 ± 11 μm^2. When mice were injected i.m. with pCK-HGF-X7, the magnitude of denervation-induced muscle loss was reduced from 61% to 41% (Figure 5B). Overall, our data show that exogenous addition of HGF, delivered in the form of a plasmid expression vector, could slow down the progress of neurogenic muscle atrophy.

The effects of i.m. injection of pCK-HGF-X7 on atrogenes were also measured. Denervation was induced, and pCK or pCK-HGF-X7 was injected i.m. into the TA. Three days after denervation, TA muscles

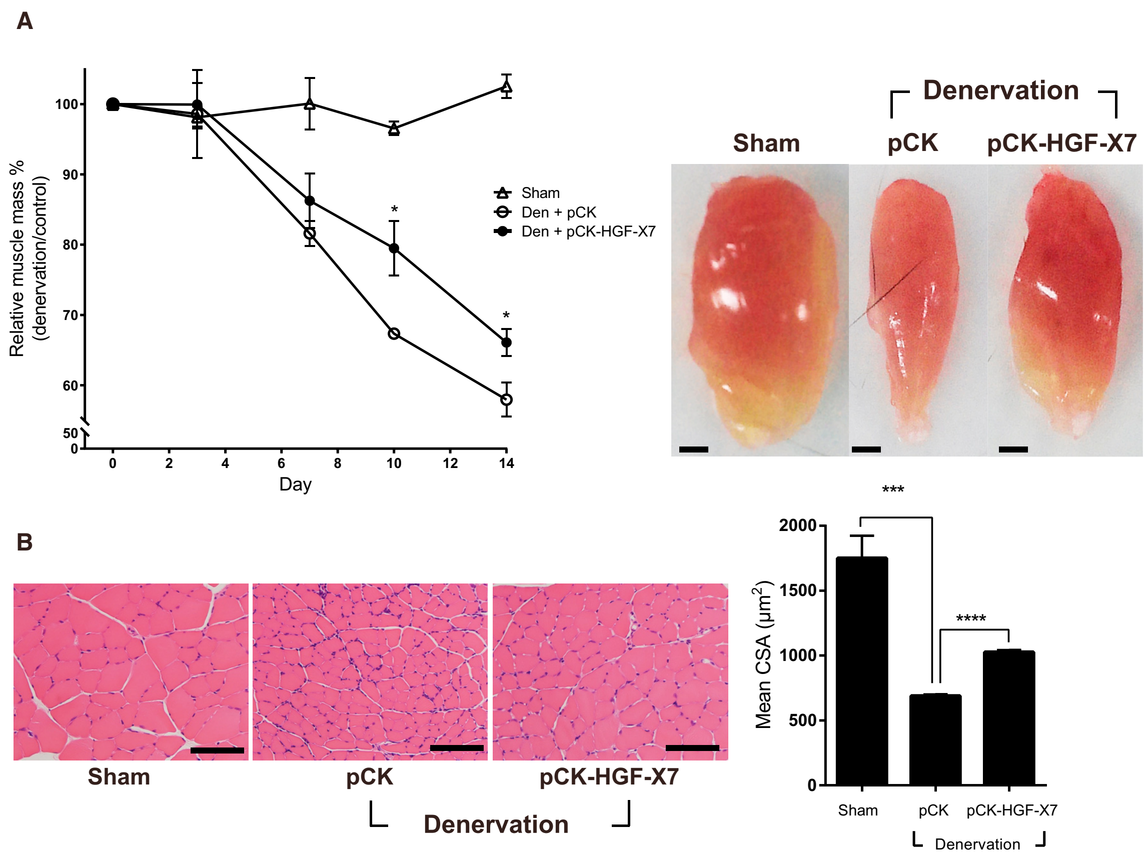

Figure 5. Effect of HGF Overexpression by Intramuscular Injection of an HGF-Expressing Plasmid on Muscle Atrophy

pCK-HGF-X7 was injected i.m. at the time of sciatic nerve transection, followed by one repeat injection 7 days later. TA muscles were prepared at appropriate time points. (A) Effect on TA muscle weight. Representative TA muscles from 14 days after denervation are shown. *p < 0.05 versus the Den+pCK group (one-way ANOVA), n = 4 per group. Scale bars, 1 mm. (B) Effect on the cross-sectional area of TA muscles. TA muscles were analyzed 10 days after denervation. At least 300 muscle fiber areas were counted per sample. Mean CSA is indicated in the graph. ***p < 0.001, ****p < 0.0001 (one-way ANOVA), n = 4 per group. Scale bars, 100 μm. All data are represented as mean ± SEM. See also Figure S3.

were isolated, and the expression level was measured using qRT-PCR. The levels of MuRF1 and Atrogin-1 were highly increased after denervation, but pCK-HGF-X7 treatment reduced the denervation-mediated induction of these genes (Figure 6A).

The effect on HDAC4 was also analyzed by measuring the RNA and protein levels 3 days after denervation and plasmid injection. Denervation greatly increased the RNA level of HDAC4, but i.m. injections of pCK-HGF-X7 had no significant effect (Figure 6B). When the protein level was measured, however, a completely different picture emerged; pCK-HGF-X7 administration significantly reduced the denervation-mediated increase in HDAC4 protein level (Figure 6C).

The effects of pCK-HGF-X7 on primary and mature miR-206 RNAs were determined by qRT-PCR. The level of miR-206 primary transcript was increased by denervation and became even higher by i.m. injection of pCK-HGF-X7 (Figure 6D). A similar observation was made regarding the level of mature miR-206 (Figure 6E). These data strongly indicate that HGF overexpression by gene transfer technology could reduce the RNA level of atrogenes by controlling miR-206 and HDAC4.

DISCUSSION

In this report, we demonstrate that HGF/c-met signaling plays a compensatory role(s) in mitigating muscle atrophy because of denervation. The HGF level was increased by 3- to 5-fold following denervation. Treating denervated mice with a specific inhibitor for c-met, PHA-665752, aggravated muscle atrophy, as measured by muscle mass and its cross-sectional area. Consistent with this observation, treatment with PHA-665752 further increased the expression level of atrogenes like MuRF1 and Atrogin-1 but reduced that of miR-206. Exogenous supply of the HGF protein to the affected region by i.m. injection of a highly efficient plasmid expression vector improved muscle atrophy by all measurements, including muscle weight, cross-sectional area, and the expression levels of miR-206, HDAC4, and atrogenes. Taken together,

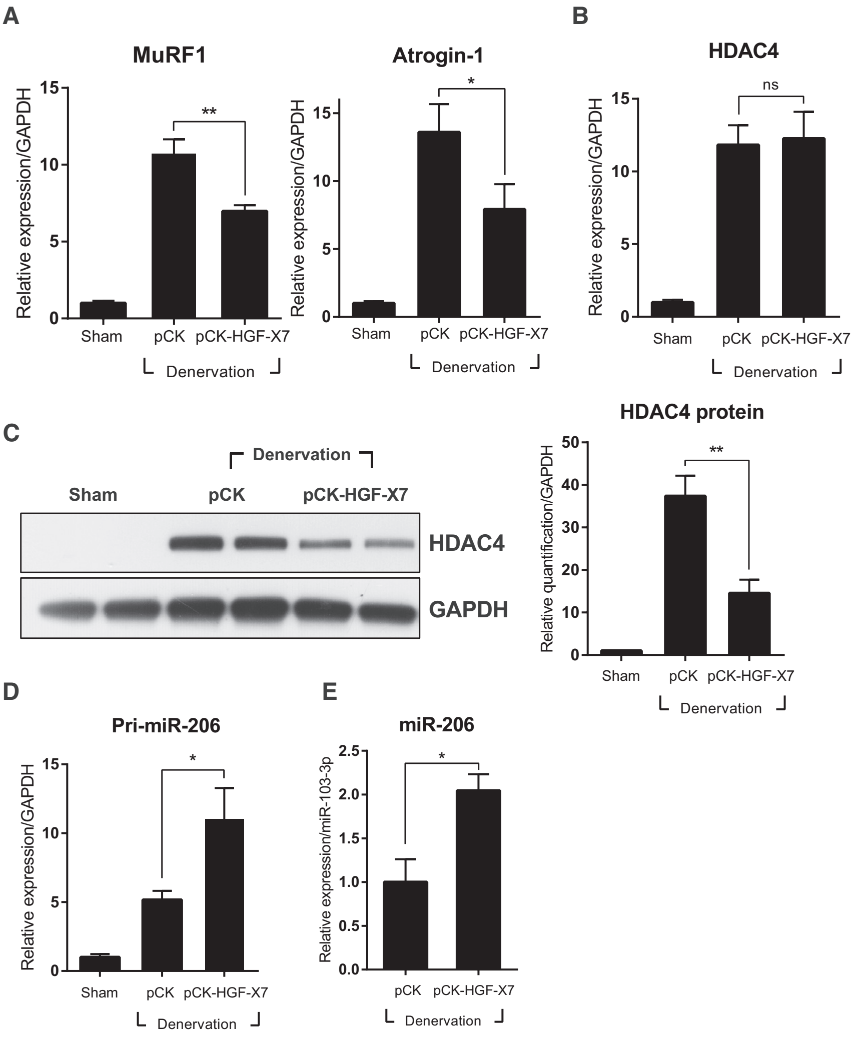

Figure 6. Effect of HGF Overexpression by Intramuscular Injection of an HGF-Expressing Plasmid on the miR-206-HDAC4 Cascade

pCK-HGF-X7 was administered i.m. at the time of sciatic nerve transection. Three days after denervation, the TA muscle was isolated, and total RNAs and proteins were analyzed by qRT-PCR and western blot. (A) Effect on the expression of MuRF1 and Atrogin-1. $^*p < 0.05$, $^{**}p < 0.01$ (one-way ANOVA), n = 4 per group. (B) Effect on HDAC4 RNA. (C) Effect on HDAC4 protein. For the western blot, two representative results are shown. Two independent experiments were performed (n = 4). Values were normalized to GAPDH for both RNA and protein analysis. $^{**}p < 0.01$ (one-way ANOVA). (D) Effect on the pri-miR-206 transcript. Values were normalized to GAPDH. $^{**}p < 0.01$ (one-way ANOVA), n = 4 per group. (E) Effect on mature miR-206. Values were normalized to miR-103a-3p. $^*p < 0.05$ (unpaired Student's t test). n = 3 per group. All data are represented as mean ± SEM.

HGF/c-met signaling appears to modulate the miR-206-HDAC4 cascade in denervated muscle.

TGF-β has been reported to downregulate the expression of miR-206 through Smad3.[26] We found that treatment of C2C12 cells with recombinant hHGF protein increased the RNA level of miR-206 while decreasing the amount of phosphorylated Smad3 protein induced by TGF-β, indicating that HGF might counteract the biological consequences generated by TGF-β. Consistently, HGF has been reported to increase the activity of TG-interacting factor (TGIF) and galectin-7, both of which act as repressors of TGF-β-stimulated signal transduction by inhibiting transcriptional activity or translocation of Smad3 from the cytoplasm to the nucleus, respectively.[35–37] These data suggest that HGF may be used as a basis for developing therapeutic agents for diseases where TGF-β is a major pathologic factor.

JNK appears to play a key role in the control by HGF of TGF-β-mediated Smad3 phosphorylation. Among several pharmacological inhibitors, SP600125, a JNK inhibitor, was the only one that could relieve the HGF-mediated suppression of Smad3 phosphorylation. Together with data from previous publications, JNK appears to control Smad3 in two ways: by transcriptional regulation of TGF-β[38] and through phosphorylation of the linker region of Smad3.[39] The former is not the case for HGF because the RNA level of TGF-β was not changed by HGF treatment in our experiments. Therefore, HGF may follow the case of epidermal growth factor (EGF), which inhibits the activity of Smad3 by phosphorylating the linker region between Mad homology-1 (MH1) and MH2 and, subsequently, suppresses phosphorylation of serine 423/425 residues at the C terminus.[40] The final outcome is a reduction in the amount of the transcriptionally active form of Smad3. It remains to be elucidated whether HGF also regulates TGF-β signaling by controlling phosphorylation of the linker region of Smad3.

Muscle atrophy results from an imbalance between synthesis and breakdown of muscle proteins. The data from our study suggest that HGF/c-met signaling might improve atrophic conditions by slowing down the breakdown process through suppression of atrogene expression. It is interesting to note a difference between our data and those by Hauerslev et al.,[18] who used the mouse hypoxia-induced muscle atrophic model. In that study, mouse recombinant HGF protein was administered i.p. once, and it was observed that the mTOR-S6K pathway was activated, and muscle protein synthesis was facilitated, within a few hours. These results suggest that the mTOR pathway might be involved in the effect of HGF on neurogenic muscle atrophy. However, mTOR seemed to play only a small role in our case. For example, inhibition of HGF/c-met signaling by daily

i.p. injection of the c-met inhibitor PHA-665752 did not affect the phosphorylation status of mTOR (Figure S1A), and mTOR inhibition did not affect the HGF-mediated upregulation of pri-miR-206 expression in C2C12 cells (Figure S2A). Taken together, HGF may work differently in these two different muscle atrophy models, each induced by hypoxia or denervation.

HGF is a growth factor binding to the c-met receptor. The interaction between the ligand and the receptor turns on a series of signaling pathways, triggering biological reactions that vary depending on the types of cells. For example, in the muscle atrophy described in this report, HGF reduced the expression of HDAC4, which facilitates disease progression, and increased the level of miR-206, which has been reported to delay ALS progression.[7] Therefore, HGF may be able to produce multiple effects in various diseases associated with muscle atrophy following denervation.

Because the HGF protein has a short half-life, gene transfer technology may provide a powerful way to deliver the HGF protein. Using naked DNA is a particularly attractive method because high-level HGF gene expression for a long term is undesirable because of its angiogenic and, thus, potentially oncogenic property.[41] All that is needed is an amount of HGF protein that can trigger reactions and then disappear, rather than lingering for a long time. In our study, pCK-HGF-X7 (VM202) seemed to be generating an amount of HGF protein sufficient to provide visible therapeutic effects. Our results are consistent with positive data observed in several clinical studies done for peripheral and coronary artery diseases and neurological diseases as well as in respective animal models involving pCK-HGF-X7.[30,31,33,34,42–46]

In summary, we demonstrated that HGF/c-met signaling could improve muscle atrophic conditions by upregulating the expression of miR-206. miR-206 is now well known to play important roles in a majority of neurogenic muscle atrophy cases, including ALS. Current treatment methods for these diseases are extremely limited; their efficacy, if any, is marginal, and safety is questioned, as in the case of riluzole or valproic acid, respectively.[47,48] Given the safety and efficacy records of pCK-HGF-X7 (VM202), shown in several clinical studies for other indications, further studies are warranted to investigate the potential of using HGF, and in particular, plasmid DNA vectors expressing HGF, for various neuromuscular diseases.

MATERIALS AND METHODS

Animal Care

Ten-week-old male C57BL/6 mice were purchased from Orient Bio (Seongnam, Korea) for animal studies. Mice were housed at 24°C with a 12-hr light-dark cycle. All experiments were performed according to the guidelines set by the International Animal Care and Use Committee at Seoul National University.

Surgical Procedures

All surgical protocols were approved by the International Animal Care and Use Committee at Seoul National University. For sciatic nerve transection, 10-week-old male C57BL/6 mice were anesthetized with isoflurane. The sciatic nerve of the right leg was cut, and a 3-mm piece was excised. To prevent nerve reattachment, severed nerve endings were tied with 6-0 black silk suture (AILEE, Pusan, Korea). Then the incision was sutured using 5-0 silk suture (AILEE, Pusan, Korea). Sham surgery was performed by following the same procedure without severing the sciatic nerve. PHA-665752 (Tocris Bioscience, MO), a c-met inhibitor, was dissolved in DMSO (Sigma-Aldrich, MO) and administered i.p. to each mouse on a daily basis with a dose of 20 mg/kg. For i.m. injection, a 0.3-mm needle size and 0.5 mL insulin syringe (BD Biosciences, NJ) were used. The pCK or pCK-HGF-X7 plasmid expression vector was dissolved in 50 μL PBS (2 μg/μL). The injection procedure was performed by injecting the needle parallel to the tibia and then delivering the plasmid into the middle of the TA muscle.

Immunohistochemistry

Immunohistochemical analyses were performed as described previously.[49] Briefly, TA muscles were fixed in 4% paraformaldehyde in PBS and cryo-sectioned to 6-μm thickness. Sections were washed in 0.1 M PBS (pH 7.4) twice and then blocked for 1 hr with PBS containing 5% fetal bovine serum (Corning Life Sciences, NY), 5% donkey serum (Jackson ImmunoResearch Laboratories, PA), 2% BSA (Sigma-Aldrich, MA), and 0.1% Triton X-100 (Sigma-Aldrich, MA). Samples were incubated with primary antibodies diluted in blocking buffer overnight at 4°C. Sections were washed four times in PBS and incubated for 1 hr at room temperature with secondary antibodies (Invitrogen, CA) diluted in PBS. Immunostained samples were further washed 6 times and counterstained with DAPI (Sigma-Aldrich, MA) for nuclear staining. The fluorescence images were obtained using a Zeiss LSM 700 confocal microscope (Zeiss, Oberkochen, Germany).

H&E Staining and Morphometric Analysis

TA muscles were fixed in 10% normalized buffered formalin (Sigma-Aldrich, MA) and dehydrated with a gradient series of ethanol from 70% to 100%. Samples were embedded in the paraffin block and sectioned to 6-μm thickness. A paraffin section of the TA muscle was stained with H&E to analyze a cross-sectional area of the muscle. The area of each myofiber was measured by ImageJ software (NIH, MD). More than 300 myofibers were assessed from 4 individual mice in each group.

RNA Isolation and qRT-PCR

TAs were prepared and mechanistically homogenized using polypropylene pestles (Bel-Art Scienceware, NJ), and total RNA was extracted in RNAiso (Takara, Kusatsu, Japan) following the manufacturer's instructions. One microgram of RNA was converted to cDNA using oligo dT primers (QIAGEN, Hilden, Germany) and Reverse Transcriptase XL (avian myeloblastosis virus [AMV]) (Takara, Kusatsu, Japan). Gene expression was assessed using real-time qPCR with the Thermal Cycler Dice Real Time System TP800 (Takara, Kusatsu, Japan) and SYBR Premix Ex Taq (Takara, Kusatsu, Japan). For miRNA analysis, RNA was converted to cDNA using the miRCURY

LNA Universal cDNA synthesis kit (Exiqon, Vedbaek, Denmark). Gene expression was measured using real-time qPCR with the ExiLENT SYBR Green Master Mix kit (Exiqon, Vedbaek, Denmark). Mature miR-206- and miR-103-3p-specific primers were purchased from Exiqon.

ELISA

TA muscles were prepared and mechanistically homogenized using polypropylene pestles (Bel-Art Scienceware, NJ), and total proteins were extracted in radioimmunoprecipitation assay (RIPA) lysis buffer (Sigma-Aldrich, MO) containing a protease inhibitor (Roche, Basel, Switzerland), phosphatase inhibitor (Roche, Basel, Switzerland), and PMSF (Sigma-Aldrich, MO). Samples were centrifuged at 12,000 rpm for 15 min at 4°C, and the supernatants containing total protein were subjected to mHGF or hHGF ELISA (R&D Systems, MN) following the manufacturer's protocol.

Western Blot

For immunoblotting, TAs were prepared and homogenized in RIPA lysis buffer (Sigma-Aldrich, MO) containing a protease inhibitor (Roche, Basel, Switzerland) and phosphatase inhibitor (Roche, Basel, Switzerland) using polypropylene pestles (Bel-Art Scienceware, NJ). Equal amounts of protein were then resolved by 10% SDS-polyacrylamide gel and transferred to polyvinylidene fluoride membranes (Millipore, MA). The membranes were blocked with 5% BSA (Gibco, MA) in TBST (1 M Tris-HCl [pH 7.4], 0.9% NaCl, and 0.1% Tween 20) for 1 hr and probed with antibodies diluted in 3% BSA blocking solution overnight at 4°C. Membranes were then incubated with HRP-conjugated anti-mouse or anti-rabbit immunoglobulin G (IgG) (1:100,000; Sigma-Aldrich, MO) for 1 hr, and the protein bands were visualized with an enhanced chemiluminescence system (Millipore, MA). Quantification of the band intensity was done by ImageJ software (NIH, MD)

Cell Culture and Reagents

C2C12 myoblasts were grown in DMEM (Welgene, Gyeongsan, Korea) supplemented with 10% fetal bovine serum (FBS) (Corning, NY) and antibiotics (100 U/mL penicillin and 100 μg/mL streptomycin; Sigma-Aldrich, MO). Cells were differentiated in DMEM supplemented with 2% horse serum (Sigma-Aldrich, MO). Recombinant hHGF (R&D Systems, MN) and recombinant TGF-β (eBioscience, MA) were used at appropriate concentrations. U0126 (an MEK1/2 inhibitor, Sigma-Aldrich, MO), SB203580 (a p38 inhibitor, Calbiochem, MA), SP600125 (a JNK inhibitor, Sigma Aldrich, MO), and Akti1/2 (an Akt inhibitor, Sigma-Aldrich, MO) were used at 10 μM, and rapamycin (an mTOR inhibitor, Sigma-Aldrich, MO) was used at 100 nM for experiments.

Statistical Analysis

All values are represented as mean ± SEM from two or more independent experiments. Statistical significance was determined using unpaired Student's t test or one-way ANOVA followed by Bonferroni's multiple comparison tests, provided by the GraphPad Prism 7 software (GraphPad, CA).

SUPPLEMENTAL INFORMATION

Supplemental Information includes three figures and one table and can be found with this article online at https://doi.org/10.1016/j.omtn.2018.06.013.

AUTHOR CONTRIBUTIONS

W.C. designed the study, performed the experiments, analyzed the data, and wrote the manuscript. Junghun Lee, Jaeman Lee, and K.R.K. conducted the experiments. S.K. designed the study and wrote the manuscript.

CONFLICTS OF INTEREST

Junghun Lee, K.R.K., and S.K. are employees or shareholders of ViroMed Co., Ltd., whose plasmid DNA (pCK-HGF-X7) was used in this work.

ACKNOWLEDGMENTS

This work was supported by research grants from the Basic Science Research Programs through the National Research Foundations of Korea (2012R1A1A2008018y) funded by the Ministry of Education and ViroMed Co., Ltd. (to S.K.).

REFERENCES

1. Beehler, B.C., Sleph, P.G., Benmassaoud, L., and Grover, G.J. (2006). Reduction of skeletal muscle atrophy by a proteasome inhibitor in a rat model of denervation. Exp. Biol. Med. (Maywood) *231*, 335–341.
2. Moresi, V., Williams, A.H., Meadows, E., Flynn, J.M., Potthoff, M.J., McAnally, J., Shelton, J.M., Backs, J., Klein, W.H., Richardson, J.A., et al. (2010). Myogenin and class II HDACs control neurogenic muscle atrophy by inducing E3 ubiquitin ligases. Cell *143*, 35–45.
3. Choi, M.-C., Cohen, T.J., Barrientos, T., Wang, B., Li, M., Simmons, B.J., Yang, J.S., Cox, G.A., Zhao, Y., and Yao, T.P. (2012). A direct HDAC4-MAP kinase crosstalk activates muscle atrophy program. Mol. Cell *47*, 122–132.
4. Kim, H.K., Lee, Y.S., Sivaprasad, U., Malhotra, A., and Dutta, A. (2006). Muscle-specific microRNA miR-206 promotes muscle differentiation. J. Cell Biol. *174*, 677–687.
5. Dey, B.K., Gagan, J., and Dutta, A. (2011). miR-206 and -486 induce myoblast differentiation by downregulating Pax7. Mol. Cell. Biol. *31*, 203–214.
6. Taulli, R., Bersani, F., Foglizzo, V., Linari, A., Vigna, E., Ladanyi, M., Tuschl, T., and Ponzetto, C. (2009). The muscle-specific microRNA miR-206 blocks human rhabdomyosarcoma growth in xenotransplanted mice by promoting myogenic differentiation. J. Clin. Invest. *119*, 2366–2378.
7. Williams, A.H., Valdez, G., Moresi, V., Qi, X., McAnally, J., Elliott, J.L., Bassel-Duby, R., Sanes, J.R., and Olson, E.N. (2009). MicroRNA-206 delays ALS progression and promotes regeneration of neuromuscular synapses in mice. Science *326*, 1549–1554.
8. Ohmichi, H., Matsumoto, K., and Nakamura, T. (1996). In vivo mitogenic action of HGF on lung epithelial cells: pulmotrophic role in lung regeneration. Am. J. Physiol. *270*, L1031–L1039.
9. Pelicci, G., Giordano, S., Zhen, Z., Salcini, A.E., Lanfrancone, L., Bardelli, A., Panayotou, G., Waterfield, M.D., Ponzetto, C., Pelicci, P.G., et al. (1995). The motogenic and mitogenic responses to HGF are amplified by the Shc adaptor protein. Oncogene *10*, 1631–1638.
10. Ding, S., Merkulova-Rainon, T., Han, Z.C., and Tobelem, G. (2003). HGF receptor up-regulation contributes to the angiogenic phenotype of human endothelial cells and promotes angiogenesis in vitro. Blood *101*, 4816–4822.
11. Xiao, G.-H., Jeffers, M., Bellacosa, A., Mitsuuchi, Y., Vande Woude, G.F., and Testa, J.R. (2001). Anti-apoptotic signaling by hepatocyte growth factor/Met via the phosphatidylinositol 3-kinase/Akt and mitogen-activated protein kinase pathways. Proc. Natl. Acad. Sci. USA *98*, 247–252.

12. Mizuno, S., Matsumoto, K., and Nakamura, T. (2001). Hepatocyte growth factor suppresses interstitial fibrosis in a mouse model of obstructive nephropathy. Kidney Int. *59*, 1304–1314.
13. Jennische, E., Ekberg, S., and Matejka, G.L. (1993). Expression of hepatocyte growth factor in growing and regenerating rat skeletal muscle. Am. J. Physiol. *265*, C122–C128.
14. Tatsumi, R., Anderson, J.E., Nevoret, C.J., Halevy, O., and Allen, R.E. (1998). HGF/SF is present in normal adult skeletal muscle and is capable of activating satellite cells. Dev. Biol. *194*, 114–128.
15. Allen, R.E., Sheehan, S.M., Taylor, R.G., Kendall, T.L., and Rice, G.M. (1995). Hepatocyte growth factor activates quiescent skeletal muscle satellite cells in vitro. J. Cell. Physiol. *165*, 307–312.
16. Sheehan, S.M., Tatsumi, R., Temm-Grove, C.J., and Allen, R.E. (2000). HGF is an autocrine growth factor for skeletal muscle satellite cells in vitro. Muscle Nerve *23*, 239–245.
17. Miller, K.J., Thaloor, D., Matteson, S., and Pavlath, G.K. (2000). Hepatocyte growth factor affects satellite cell activation and differentiation in regenerating skeletal muscle. Am. J. Physiol. Cell Physiol. *278*, C174–C181.
18. Hauerslev, S., Vissing, J., and Krag, T.O. (2014). Muscle atrophy reversed by growth factor activation of satellite cells in a mouse muscle atrophy model. PLoS ONE *9*, e100594.
19. Sugiura, T., Kawaguchi, Y., Soejima, M., Katsumata, Y., Gono, T., Baba, S., Kawamoto, M., Murakawa, Y., Yamanaka, H., and Hara, M. (2010). Increased HGF and c-Met in muscle tissues of polymyositis and dermatomyositis patients: beneficial roles of HGF in muscle regeneration. Clin. Immunol. *136*, 387–399.
20. Wong, V., Glass, D.J., Arriaga, R., Yancopoulos, G.D., Lindsay, R.M., and Conn, G. (1997). Hepatocyte growth factor promotes motor neuron survival and synergizes with ciliary neurotrophic factor. J. Biol. Chem. *272*, 5187–5191.
21. Sun, W., Funakoshi, H., and Nakamura, T. (2002). Overexpression of HGF retards disease progression and prolongs life span in a transgenic mouse model of ALS. J. Neurosci. *22*, 6537–6548.
22. Gurney, M.E., Pu, H., Chiu, A.Y., Dal Canto, M.C., Polchow, C.Y., Alexander, D.D., Caliendo, J., Hentati, A., Kwon, Y.W., Deng, H.X., et al. (1994). Motor neuron degeneration in mice that express a human Cu,Zn superoxide dismutase mutation. Science *264*, 1772–1775.
23. Rao, P.K., Kumar, R.M., Farkhondeh, M., Baskerville, S., and Lodish, H.F. (2006). Myogenic factors that regulate expression of muscle-specific microRNAs. Proc. Natl. Acad. Sci. USA *103*, 8721–8726.
24. Sweetman, D., Goljanek, K., Rathjen, T., Oustanina, S., Braun, T., Dalmay, T., and Münsterberg, A. (2008). Specific requirements of MRFs for the expression of muscle specific microRNAs, miR-1, miR-206 and miR-133. Dev. Biol. *321*, 491–499.
25. Ozawa, J., Kurose, T., Kawamata, S., Kaneguchi, A., Moriyama, H., and Kito, N. (2013). Regulation of connective tissue remodeling in the early phase of denervation in a rat skeletal muscle. Biomed. Res. *34*, 251–258.
26. Winbanks, C.E., Wang, B., Beyer, C., Koh, P., White, L., Kantharidis, P., and Gregorevic, P. (2011). TGF-β regulates miR-206 and miR-29 to control myogenic differentiation through regulation of HDAC4. J. Biol. Chem. *286*, 13805–13814.
27. Mizuno, S., and Nakamura, T. (2004). Suppressions of chronic glomerular injuries and TGF-β 1 production by HGF in attenuation of murine diabetic nephropathy. Am. J. Physiol. Renal Physiol. *286*, F134–F143.
28. Dong, Y., Lakhia, R., Thomas, S.S., Dong, Y., Wang, X.H., Silva, K.A.S., and Zhang, L. (2013). Interactions between p-Akt and Smad3 in injured muscles initiate myogenesis or fibrogenesis. Am. J. Physiol. Endocrinol. Metab. *305*, E367–E375.
29. Kawaida, K., Matsumoto, K., Shimazu, H., and Nakamura, T. (1994). Hepatocyte growth factor prevents acute renal failure and accelerates renal regeneration in mice. Proc. Natl. Acad. Sci. USA *91*, 4357–4361.
30. Pyun, W.B., Hahn, W., Kim, D.S., Yoo, W.S., Lee, S.D., Won, J.H., Rho, B.S., Park, Z.Y., Kim, J.M., and Kim, S. (2010). Naked DNA expressing two isoforms of hepatocyte growth factor induces collateral artery augmentation in a rabbit model of limb ischemia. Gene Ther. *17*, 1442–1452.
31. Hahn, W., Pyun, W.B., Kim, D.S., Yoo, W.S., Lee, S.D., Won, J.H., Shin, G.J., Kim, J.M., and Kim, S. (2011). Enhanced cardioprotective effects by coexpression of two isoforms of hepatocyte growth factor from naked plasmid DNA in a rat ischemic heart disease model. J. Gene Med. *13*, 549–555.
32. Cho, K.R., Choi, J.-S., Hahn, W., Kim, D.S., Park, J.S., Lee, D.S., and Kim, K.B. (2008). Therapeutic angiogenesis using naked DNA expressing two isoforms of the hepatocyte growth factor in a porcine acute myocardial infarction model. Eur. J. Cardiothorac. Surg. *34*, 857–863.
33. Kessler, J.A., Smith, A.G., Cha, B.S., Choi, S.H., Wymer, J., Shaibani, A., Ajroud-Driss, S., and Vinik, A.; VM202 DPN-II Study Group (2015). Double-blind, placebo-controlled study of HGF gene therapy in diabetic neuropathy. Ann. Clin. Transl. Neurol. *2*, 465–478.
34. Kibbe, M.R., Hirsch, A.T., Mendelsohn, F.O., Davies, M.G., Pham, H., Saucedo, J., Marston, W., Pyun, W.B., Min, S.K., Peterson, B.G., et al. (2016). Safety and efficacy of plasmid DNA expressing two isoforms of hepatocyte growth factor in patients with critical limb ischemia. Gene Ther. *23*, 306–312.
35. Inagaki, Y., Higashi, K., Kushida, M., Hong, Y.Y., Nakao, S., Higashiyama, R., Moro, T., Itoh, J., Mikami, T., Kimura, T., et al. (2008). Hepatocyte growth factor suppresses profibrogenic signal transduction via nuclear export of Smad3 with galectin-7. Gastroenterology *134*, 1180–1190.
36. Dai, C., and Liu, Y. (2004). Hepatocyte growth factor antagonizes the profibrotic action of TGF-β1 in mesangial cells by stabilizing Smad transcriptional corepressor TGIF. J. Am. Soc. Nephrol. *15*, 1402–1412.
37. Yang, J., Dai, C., and Liu, Y. (2003). Hepatocyte growth factor suppresses renal interstitial myofibroblast activation and intercepts Smad signal transduction. Am. J. Pathol. *163*, 621–632.
38. Ventura, J.-J., Kennedy, N.J., Flavell, R.A., and Davis, R.J. (2004). JNK regulates autocrine expression of TGF-β1. Mol. Cell *15*, 269–278.
39. Li, Q., Liu, G., Yuan, H., Wang, J., Guo, Y., Chen, T., Zhai, R., Shao, D., Ni, W., and Tai, G. (2015). Mucin1 shifts Smad3 signaling from the tumor-suppressive pSmad3C/p21(WAF1) pathway to the oncogenic pSmad3L/c-Myc pathway by activating JNK in human hepatocellular carcinoma cells. Oncotarget *6*, 4253–4265.
40. Wang, G., Matsuura, I., He, D., and Liu, F. (2009). Transforming growth factor-β-inducible phosphorylation of Smad3. J. Biol. Chem. *284*, 9663–9673.
41. Bussolino, F., Di Renzo, M.F., Ziche, M., Bocchietto, E., Olivero, M., Naldini, L., Gaudino, G., Tamagnone, L., Coffer, A., and Comoglio, P.M. (1992). Hepatocyte growth factor is a potent angiogenic factor which stimulates endothelial cell motility and growth. J. Cell Biol. *119*, 629–641.
42. Carlsson, M., Osman, N.F., Ursell, P.C., Martin, A.J., and Saeed, M. (2008). Quantitative MR measurements of regional and global left ventricular function and strain after intramyocardial transfer of VM202 into infarcted swine myocardium. Am. J. Physiol. Heart Circ. Physiol. *295*, H522–H532.
43. Perin, E.C., Silva, G.V., Vela, D.C., Zheng, Y., Baimbridge, F., Gahremanpour, A., Quan, X., Hahn, W., Kim, J., Wood, K., and Kitamura, M. (2011). Human hepatocyte growth factor (VM202) gene therapy via transendocardial injection in a pig model of chronic myocardial ischemia. J. Card. Fail. *17*, 601–611.
44. Gu, Y., Zhang, J., Guo, L., Cui, S., Li, X., Ding, D., Kim, J.M., Ho, S.H., Hahn, W., and Kim, S. (2011). A phase I clinical study of naked DNA expressing two isoforms of hepatocyte growth factor to treat patients with critical limb ischemia. J. Gene Med. *13*, 602–610.
45. Kim, J.S., Hwang, H.Y., Cho, K.R., Park, E.A., Lee, W., Paeng, J.C., Lee, D.S., Kim, H.K., Sohn, D.W., and Kim, K.B. (2013). Intramyocardial transfer of hepatocyte growth factor as an adjunct to CABG: phase I clinical study. Gene Ther. *20*, 717–722.
46. Ajroud-Driss, S., Christiansen, M., Allen, J.A., and Kessler, J.A. (2013). Phase 1/2 open-label dose-escalation study of plasmid DNA expressing two isoforms of hepatocyte growth factor in patients with painful diabetic peripheral neuropathy. Mol. Ther. *21*, 1279–1286.
47. Groeneveld, G.J., Van Kan, H.J., Kalmijn, S., Veldink, J.H., Guchelaar, H.-J., Wokke, J.H., and Van den Berg, L.H. (2003). Riluzole serum concentrations in patients with ALS: associations with side effects and symptoms. Neurology *61*, 1141–1143.
48. Tong, V., Teng, X.W., Chang, T.K., and Abbott, F.S. (2005). Valproic acid II: effects on oxidative stress, mitochondrial membrane potential, and cytotoxicity in glutathione-depleted rat hepatocytes. Toxicol. Sci. *86*, 436–443.
49. Ahn, J., Jang, J., Choi, J., Lee, J., Oh, S.-H., Lee, J., Yoon, K., and Kim, S. (2014). GSK3β, but not GSK3α, inhibits the neuronal differentiation of neural progenitor cells as a downstream target of mammalian target of rapamycin complex1. Stem Cells Dev. *23*, 1121–1133.

OMTN, Volume *12*

Supplemental Information

Hepatocyte Growth Factor Regulates the miR-206-HDAC4 Cascade to Control Neurogenic Muscle Atrophy following Surgical Denervation in Mice

Wooshik Choi, Junghun Lee, Jaeman Lee, Kyeong Ryang Ko, and Sunyoung Kim

Figure S1, related to Figure 3

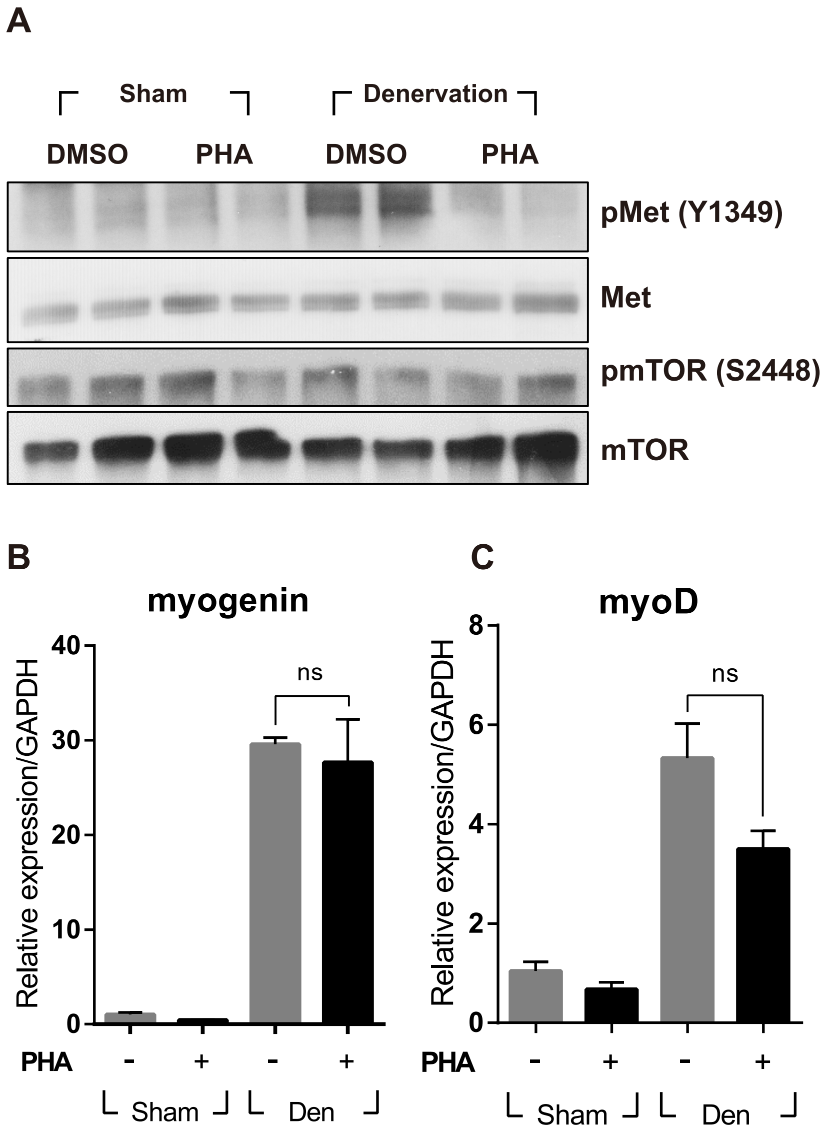

Figure S1. Effect of c-met inhibitor, PHA-665752 on c-met, mTOR phosphorylation and E-box transcription factors. After denervation by sciatic nerve transection, mice were intraperitoneally injected with 20 mg/kg of PHA-665752 on a daily basis until sacrificed. Three days later, TA muscles were prepared and total RNAs and proteins were isolated followed by RT-qPCR and Western blot respectively. (A) Effect on total and phosphorylated c-met, mTOR protein. (B) Effect on myogenin RNA. (C) Effect on myoD RNA. Values were normalized to GAPDH. ns=not significant. n=4 per group.

Figure S2, related to Figure 4

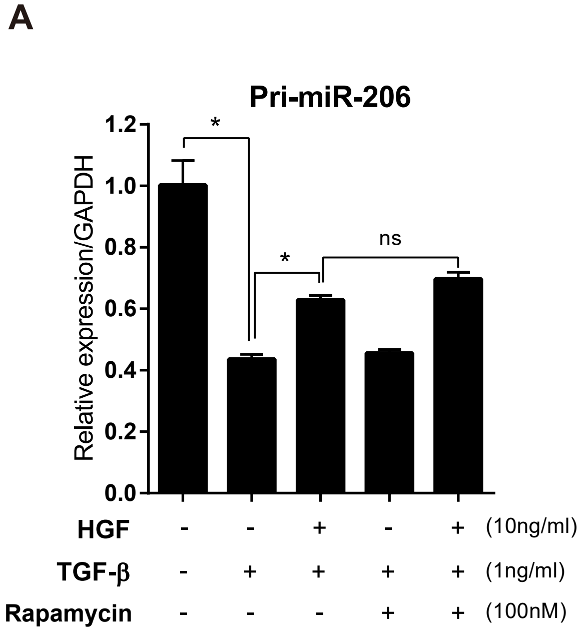

Figure S2. Effect of mTOR in HGF-mediated regulation of miR-206 expression in C2C12 cells.
C2C12 cells were plated and cultured in differentiation medium in the presence or absence of recombinant TGF-β and HGF proteins. Total RNAs were prepared and analyzed for miR-206 primary transcript. (A) Effect of mTOR inhibitor on the HGF-mediated regulation of pri-miR-206 transcript expression. Values were normalized to GAPDH. ns=not significant. *$p<0.05$, (unpaired student's t test), n=2 per group.

Figure S3, related to Figure 5

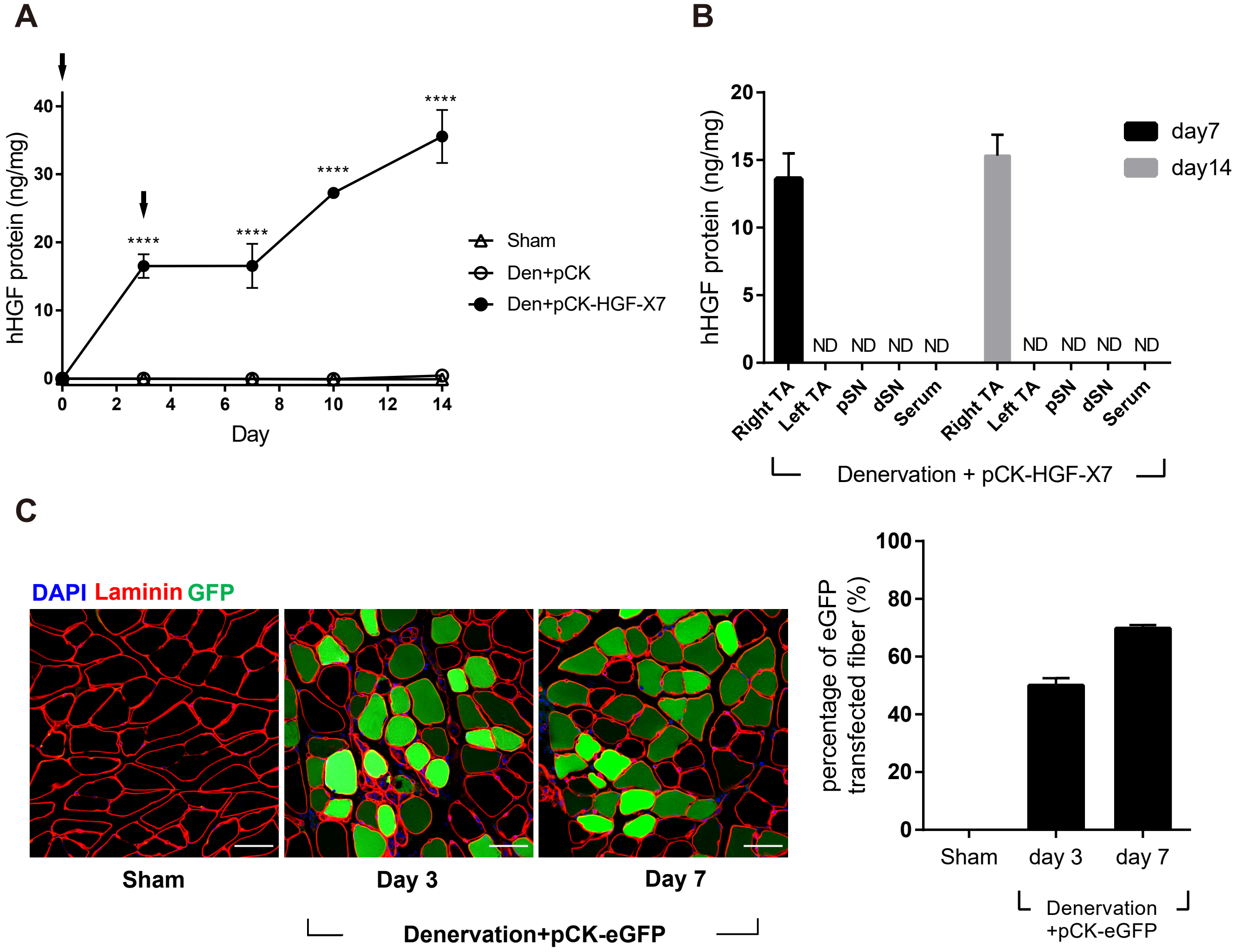

Figure S3. hHGF expression by pCK-HGF-X7 in denervated mice.
pCK-HGF-X7 was i.m. injected at the time(day 0) of sciatic nerve transection followed by one more injection on day 7. (A) Expression kinetics of the hHGF protein after denervation. Two arrows indicate i.m. injection of pCK-HGF-X7 on days 0 and 7. The muscle was isolated at 3, 7, 10, and 14 days after denervation, and total proteins were analyzed by ELISA to measure the protein level of human HGF. *p<0.05, **p<0.01, ***p<0.001, ****p<0.0001 versus pCK-treated muscle (unpaired student's t test), n=4 per group. (B) Expression kinetics of hHGF protein in TA, proximal and distal sciatic nerve, and serum. ND=not detected, n=2 per group. (C) Transfection efficiency of pCK-eGFP in denervated muscle. pCK-eGFP was i.m. injected at the time of sciatic nerve transection. TAs were costained with GFP and the membrane marker laminin (red). n=3 per group. Scale bars=50μm.

Table S1

A

Primers	sequence
mHGF_F	5'-ATCCACGATGTTCATGAGAG-3'
mHGF_R	5'-GCTGACTGCATTTCTCATTC-3'
MURF1_F	5′-TGCCTGGAGATGTTTACCAAGC-3′
MURF1_R	5′-AAACGACCTCCAGACATGGACA-3'
Atrogin-1_F	5′-AAGGCTGTTGGAGCTGATAGCA-3′
Atrogin-1_R	5′-CACCCACATGTTAATGTTGCCC-3′
Pri-miR-206_F	5'-ACCCAGTGCCCTGTGTTCCCA-3'
Pri-miR-206_R	5'-AGCGCCTCTTCTCGGTTTCCCT-3'
HDAC4_F	5'-CACACCTCTTGGAGGGTACAA-3'
HDAC4_R	5'-AGCCCATCAGCTGTTTTGTC-3'
MyoD_F	5'-CCACTCCGGGACATAGACTTG-3'
MyoD_R	5'-AAAAGCGCAGGTCTGGTGAG-3'
Myogenin_F	5'-GAGACATCCCCCTATTTCTACCA-3'
Myogenin_R	5'-GCTCAGTCCGCTCATAGCC-3'
GAPDH_F	5'-CTGGAAAGCTGTGGCGTGAT-3'
GAPDH_R	5'-CCAGGCGGCACGTCAGATCC-3'

B

Antibody	host	company	catalog	Notes
pMet (Y1349)	rabbit	Cell signaling	#3133	WB, 1:500
Met	rabbit	Sigma Aldrich	SAB4300599	WB, 1:2000
HDAC4	mouse	Cell signaling	#5392	WB, 1:1000
pSmad3 (S423/425)	rabbit	Cell signaling	#9520	WB, 1:1000
Smad3	rabbit	Cell signaling	#9523	WB, 1:1000
Laminin	rabbit	Sigma Aldrich	L9393	IHC, 1:1000
GFP	mouse	Millipore	MAB3580	IHC, 1:500

Table S1. Primers and antibodies used in this study.
(A) Primer information for RT-qPCR analysis. (B) Antibody information for Western blot hybridization and Immunohistochemistry.
